# Structural Analysis of Joints Made of Titanium Alloy TI-6AL-4V and Stainless Steel AISI 321 with Developed Conical Contact Surfaces Obtained by Diffusion Welding

**DOI:** 10.3390/ma18153596

**Published:** 2025-07-31

**Authors:** Olena Karpovych, Ivan Karpovych, Oleksii Fedosov, Denys Zhumar, Yevhen Karakash, Miroslav Rimar, Jan Kizek, Marcel Fedak

**Affiliations:** 1 Department of Rocket and Space and Innovative Technologies, Oles Honchar Dnipro National University, Nauky Ave., 72, 49005 Dnipro, Ukraine; kelvladmail@gmail.com (O.K.); ivkarp70@gmail.com (I.K.); zhumar82@gmail.com (D.Z.); 2 Department of Technology of Aircraft Manufacturing, Oles Honchar Dnipro National University, Nauky Ave., 72, 49005 Dnipro, Ukraine; fedosov.fav@gmail.com; 3 Industrial and Business Technologies, Ukrainian State University of Science and Technologies, Lazaryana Street 2, 49010 Dnipro, Ukraine; yevgenkarakash@gmail.com; 4 Department of Process Technique, Faculty of Manufacturing Technologies with a seat in Presov, Technical University of Kosice, Bayerova 1, 080 01 Presov, Slovakia; miroslav.rimar@tuke.sk (M.R.); marcel.fedak@tuke.sk (M.F.)

**Keywords:** bimetallic joints, dissimilar metals, diffusion welding, dislocation, microstructure, micro-X-ray spectral analysis

## Abstract

The object of this study is welded joints of AISI 321 and Ti-6Al-4V, obtained by diffusion welding on developed conical surfaces. The problem of creating bimetallic joints of AISI 321 and Ti-6Al-4V with developed conical contact surfaces, using diffusion welding through an intermediate Electrolytic Tough Pitch Copper (Cu-ETP) copper layer, was solved. The joints were studied using micro-X-ray spectral analysis, microstructural analysis, and mechanical tests. High mutual diffusion of copper and titanium, along with increased concentrations of Cr and V in copper, was detected. The shear strength of the obtained welded joints is 250 MPa and 235 MPa at 30 min and 15 min, respectively, which is higher than the copper layer’s strength (180 MPa). The obtained results are explained by the dislocation diffusion mechanism in the volume of grains and beyond, due to thermal deformations during welding. Under operating conditions of internal pressure and cryogenic temperatures, the strength of the connection is ensured by the entire two-layer structure, and tightness is ensured by a vacuum-tight diffusion connection. The obtained strength of the connection (250 MPa) is sufficient under the specified operating conditions. Analysis of existing solutions in the literature review indicates that industrial application of technology for manufacturing bimetallic adapters from AISI 321 stainless steel and Ti-6Al-4V titanium alloy is limited to butt joints with small geometric dimensions. Studies of the transition zone structure and diffusion processes in bimetallic joints with developed conical contact surfaces enabled determination of factors affecting joint structure and diffusion coefficients. The obtained bimetallic adapters, made of Ti-6Al-4V titanium alloy and AISI 321 stainless steel, can be used to connect titanium high-pressure vessels with stainless steel pipelines.

## 1. Introduction

Bimetallic bonds made of dissimilar metals such as titanium alloy Ti-6Al-4V and stainless steel AISI 321 are widely used in the aviation, nuclear, and chemical industries, among many others. In particular, spacecraft vessels use high-pressure tanks made of titanium alloy Ti-6Al-4V, which are connected to a pipeline made of stainless steel AISI 321. Tanks are used to store fuel components and gases in a liquid state, so the adapter that connects the tank and the pipeline is at cryogenic temperature and under the influence of an aggressive environment [1,2]. Due to installation constraints, the geometry of the tank is adapted to meet the required volume of the specific system. This adaptation typically involves optimizing the tank’s shape to fit within confined spaces while preserving mechanical strength and fluid capacity. Examples of such tank geometries are published, for instance, in the literature [2,3]. These tanks, intended for hydrazine systems in spacecraft, are constructed from titanium alloy Ti-6Al-4V and stainless steel AISI 321. The configurations include spherical geometry or a combination of spherical or elliptical domes with optional cylindrical sections of varying length, designed to optimize volume and structural integrity under cryogenic conditions.

Such operating conditions impose high operational requirements on the adapter, which include strength, tightness, corrosion resistance, and minimal weight. According to current manufacturing practices, the blanks for such adapters are produced based on the end-use configuration, with the required operational properties ensured by a relatively large wall thickness. At low temperatures, tangential stresses arise in the connection zone of such adapters due to differences in the physical and mechanical properties of the titanium alloy Ti-6Al-4V and stainless steel AISI 321, particularly their coefficients of linear thermal expansion. The magnitude of these stresses depends on the wall thickness and, at certain values, leads to the destruction of the connection.

Obtaining joints between the titanium alloy Ti-6Al-4V and stainless steel AISI 321 with the required physical and mechanical properties is a complex technological task, with various welding and brazing methods used to solve it. Numerous studies have been conducted in which methods such as fusion welding, solid-phase welding, and brazing were used to join stainless steel and titanium alloys. Each of these methods requires the use of special technological techniques and careful observance of process parameters. The most common strategy for improving the strength of joints between titanium alloy Ti-6Al-4V and stainless steel AISI 321 is to use intermediate materials in the transition layer. These layers block the diffusion of carbon from steel into the titanium alloy and form a number of solutions in a certain range of element concentrations on both sides of the joint. Intermediate layers also help reduce internal stresses in the joint and prevent its destruction. In this case, the strength of the joints is lower than the properties of the base materials and depends on the strength and thickness of the intermediate layers.

Connections obtained according to the scheme at the ends of pipe blanks are limited in their size and operating temperature range. Therefore, the use of a connection scheme with developed conical contact surfaces, as well as taking into account the difference in the thermophysical properties of the Ti-6Al-4V alloy and AISI 321 steel, will allow a connection with an expanded range of geometric and operational characteristics to be obtained. The transition layer of joints with developed conical contact surfaces is currently insufficiently studied, and scientific research on this topic is important. The research results will allow us to determine the dependence of the structure and mechanical properties of joints made of titanium alloy Ti-6Al-4V and stainless steel AISI 321 with developed conical contact surfaces on the diffusion welding modes. In practical terms, this will allow us to manufacture adapters for connecting titanium high-pressure vessels with a stainless steel pipeline.

Therefore, studies devoted to determining the structure of the transition layer and diffusion processes in bimetallic joints made of titanium alloy Ti-6Al-4V and stainless steel AISI 321 with developed conical contact surfaces are relevant.

### 1.1. State of the Art

Solid-state metal joining methods include diffusion welding, friction welding, and explosive welding. In [4], diffusion welding was used to join AISI 321 stainless steel to the titanium alloy Ti-6Al-4V using a Cu-Zn foil as an interlayer. The authors evaluated the influence of welding time (30, 45, and 60 min) at 900 °C and 2 MPa pressure on the microstructural and mechanical behavior of the joints. The results showed the presence of intermetallic compounds such as CuTi_2_, CuTi, FeTi, Fe_22_Zn_78_, and TiZn_3_ in the joint. It was also found that increasing the holding time led to a decrease in joint strength from 281 MPa to 174 MPa. The research was conducted on flat specimens using a specialized diffusion welding setup that ensured the required contact pressure and maintained a vacuum environment to protect the highly reactive Ti-6Al-4V alloy from oxidation. The reduced strength was attributed to the formation of brittle intermetallics in the transition zone. The Cu-Zn foil used as the interlayer cannot be applied in the production of adapters operating under harsh service conditions. To manufacture joints from flat and cylindrical billets, more widely used and technologically advanced fusion welding methods can be employed.

Such results are presented in [5]. The authors describe key characteristics of the transition zone’s formation and structure in joints between titanium alloys and stainless steel. The authors note that obtaining joints with the desired properties between titanium alloys and stainless steel by fusion welding is quite challenging due to the formation of brittle intermetallics, such as FeTi and Fe_2_Ti. To address this issue, the use of an interlayer material with a lower melting point than the base metals is proposed.

An example of such a joint between AMS 4911L (Ti-6Al-4V) and AISI 316L is presented in [6]. The authors employed the cold metal transfer (CMT) welding method with CuSi-3 filler wire. The resulting joints exhibited two intermetallic layers near the base metals. The hardness of these layers was higher than that of the rest of the weld. Tensile tests revealed a maximum joint strength of 200 MPa, which is significantly lower than the strength of the base metals. This method can be used for welding both flat and cylindrical billets. However, the issue of a filler material that can prevent intermetallic formation in the transition zone remains unresolved. The high temperatures and hydrodynamic effects associated with fusion welding may prevent the formation of intermetallic-free joints. Consequently, this method is not considered promising for producing bimetallic joints between titanium alloys and stainless steel. The formation of intermetallics may be avoided by welding without melting and using interlayers that form solid solutions with the base metals. This approach was employed in [7].

The authors of [7] studied joints between Ti-1.2ASN (Ti-0.5Al-0.45Si-0.2Nb) alloy and 436 stainless steel, produced by explosive welding with a pure niobium (Nb) interlayer. Thin sheets with thicknesses of 1 mm and 1.2 mm were employed, with the niobium foil having a thickness of 0.1 mm. The authors reported that after prolonged thermal exposure at 300–600 °C for 1.5 h, the microstructure of titanium–stainless steel (Ti-SS) and titanium–niobium–stainless steel (Ti-Nb-SS) welds remained stable. The paper also provides preliminary recommendations for manufacturing and applying such joints but states that the research is still ongoing. This method requires a special laser setup with complex process parameter control and a vacuum or protective gas atmosphere to avoid oxidation. As it can only produce joints from flat billets of limited thickness, it is not suitable for the manufacture of adapters between titanium alloys and stainless steel. Welding thick-walled cylindrical parts is possible using diffusion welding, friction welding, explosive welding, or hot isostatic pressing, as proposed in [8,9,10,11].

In [8], hot isostatic pressing (HIP) technology was proposed for the simultaneous joining of dissimilar materials—SS321 stainless steel with Inconel 718 and Ti-6Al-4V titanium alloy. Cylindrical billets with a high length-to-diameter ratio were used. The HIP process lasted up to 3 h. Diffusion bonding via HIP requires precise preparation of contact surfaces and the use of nickel or tantalum interlayers. The authors reported defect-free joints, achieving strengths of 460 MPa with a nickel interlayer and 680 MPa with a tantalum interlayer. This method enabled the joining of SS321 stainless steel with Ti-6Al-4V using extended cylindrical surfaces. The main challenges include the long process duration, the need for specialized HIP equipment, and the complexity of placing the interlayer between cylindrical surfaces.

In [9], the authors conducted comparative studies of diffusion welding and friction welding between Ti-6Al-4V and 304L stainless steel using copper as an interlayer. They reported that friction welding produced stronger joints with a shorter processing time than diffusion welding. End-to-end welding of cylindrical specimens was performed. Friction welding achieved a joint strength of 520 MPa, in comparison to 282 MPa for diffusion welding. The friction welding process required 36 min, compared to 127 min for diffusion welding. This method enables high-strength joints in relatively short processing times. However, the authors did not provide data on the joint’s ductility or its performance under elevated or reduced temperatures and internal pressure. Considering the property differences between Ti-6Al-4V and 304L under such conditions, internal stresses may arise in the joint. These stresses increase with diameter, potentially leading to failure. The use of friction stir welding (FSW) in a water bath was investigated by the authors of [10] to obtain joints created by layering Ti-6Al-4V and Al-4Cu-1Mg at various FSW tool rotation speeds. The authors aimed to find the most suitable rotation speeds that would lead to the plasticization of the titanium alloy, reduce defects in the mixing zone, and achieve a better bond with the aluminum alloy. However, increasing the FSW tool rotation speed led to an increased formation of intermetallic compounds (IMCs). The authors of [11] adopted a different approach from conventional FSW techniques when joining Ti-6Al-4V and the aluminum alloy AA7075-T651, aiming to avoid the formation of intermetallic compounds (IMCs). Pre-drilled holes were created in the titanium alloy, and the joint was achieved through mechanical interlocking, whereby the aluminum material deforms into these holes in the titanium plate, similar to in a riveting process. Therefore, friction welding requires further research and real-world validation.

In [12], explosion welding was employed to join pure titanium (TP 270C) and SUS 821L1 stainless steel. Flat billets with a thickness of 3 mm were employed. Some joints achieved a strength exceeding 700 MPa but exhibited reduced ductility compared to the base metals. This method requires complex equipment, specialized testing facilities, and thorough preparation of both the assembly and the contact surfaces. Additionally, explosion welding presents significant safety hazards. The lower ductility can cause sudden, unpredictable joint failure, requiring further technological development.

In [13], diffusion welding between titanium alloy (Ti-6Al-4V) and stainless steel (AISI 321) using mating conical surfaces was studied. Components were assembled coaxially using cylindrical or conical surfaces, with welding pressure generated by the difference in coefficients of thermal expansion (CTEs) of the materials. Microstructural and micro-X-ray spectral analysis were employed to plot concentration profiles of the main and alloying elements, revealing that welding temperature and holding time were key factors affecting the width of the transition zone and the formation rate of intermetallics. Diffusion in the Ti-6Al-4V—AISI 321 joints followed a dislocation mechanism. The joint strength was approximately 63.46 MPa, significantly lower than the 330–380 MPa shear strength of AISI 321 stainless steel. Notably, welding was performed in air using a conventional muffle furnace to achieve the required temperature. The required pressure on the contact surfaces was achieved owing to the difference in CTEs, enabling fast diffusion processes. That is, this method does not require the use of special, expensive equipment with a special atmosphere or vacuum.

A comparative analysis of the methods described in [8,9,10,11,12,13] for producing bimetallic joints between stainless steel and titanium alloys reveals several key features:

- Use of high-cost specialized equipment;
- Meticulous preparation of contact surfaces;
- Use of interlayers between base metals.

The highest joint strength—matching that of the weaker stainless steel base metal—was achieved only by explosive welding. However, such joints exhibit reduced ductility, and the process itself is complex, is costly, and presents significant safety hazards. Most studies were conducted on flat, thin-walled specimens or cylindrical billets joined via flat end surfaces. Such bimetallic billets may be used to produce adapters with small geometric dimensions.

To overcome these limitations, diffusion welding between Ti-6Al-4V and AISI 321 via developed conical surfaces is proposed, as reported in [13]. To avoid the formation of intermetallics in the transition zone, the authors propose studying diffusion welding using a copper interlayer.

The above issues justify conducting research on diffusion welding between Ti-6Al-4V and AISI 321 billets via extended conical contact surfaces using a Cu-ETP interlayer. This welding scheme will utilize the thermophysical properties of the materials to generate the required contact pressure during welding and enhance joint strength under cryogenic service conditions.

### 1.2. Aim of This Work

The objective of this article is to analyze diffusion parameters and the structure of transition zones based on the technological conditions of diffusion bonding for AISI 321–Cu–Ti-6Al-4V joints with developed conical contact surfaces through an intermediate copper layer of Cu-ETP. The obtained results will enable the production of bimetallic adapters made of Ti-6Al-4V titanium alloy and AISI 321 stainless steel. The adapters will feature the required mechanical and thermal operational properties to connect high-pressure titanium vessels to stainless steel pipelines.

To achieve this goal, the following tasks were set:

- Based on microstructural and micro-X-ray spectral analysis data, determine the diffusion depth of base and alloying elements in the AISI 321–Cu–Ti-6Al-4V joint;
- Based on the diffusion depth, determine the diffusion coefficients of the elements in the AISI 321–Cu–Ti-6Al-4V joint and the diffusion mechanisms;
- Based on mechanical testing results, assess the influence of holding time on the strength of the AISI 321–Cu–Ti-6Al-4V joint and determine the optimal technological parameters for diffusion bonding.

## 2. Materials and Methods

This study focuses on welded joints between AISI 321 and Ti-6Al-4V with a Cu-ETP interlayer, obtained by diffusion bonding along developed conical surfaces.

The material used for welding was the titanium alloy Ti-6Al-4V, whose properties are provided by its manufacturers, as referenced in [14,15].

In particular, this study focuses on the characteristics of the Ti-6Al-4V alloy used in the welding process. Its chemical composition and fundamental physical–mechanical and thermophysical properties—according to data reported in [14]—are summarized in Table 1, Table 2 and Table 3.

The second material used in the welding process and examined in this study was the austenitic stainless steel AISI 321, whose properties are provided by its manufacturers, as referenced in [16,17].

In [18], the authors investigated the properties of AISI 321 stainless steel, and the results of their study confirmed its good weldability, favorable cold-forming capability, and reliable mechanical properties. These findings further support the suitability of the selected steel for use in this study.

This study primarily focuses on the properties of AISI 321 steel used in the welding process. Its chemical composition, key physical–mechanical characteristics, and thermophysical properties—based on the data presented in [16]—are summarized in Table 4, Table 5 and Table 6.

Copper CU-ETP was used as the interlayer material. Its chemical composition, fundamental physical–mechanical characteristics, and thermophysical properties—based on the data presented in [19]—are summarized in Table 7, Table 8 and Table 9.

A comparative analysis of the characteristics of the Ti-6Al-4V titanium alloy and AISI 321 stainless steel showed that the steel has lower strength and a coefficient of thermal expansion (CTE) approximately twice that of the titanium alloy. Therefore, to achieve the required contact pressure at the welding temperature, the inner part can be made of stainless steel, and the outer part of titanium alloy. However, during cooling to room temperature, radial stresses will arise in the joint, which may lead to failure. If the inner part is made of titanium alloy and the outer of stainless steel, and the entire assembly is placed in an outer sleeve with a lower CTE and high strength, then upon heating to the welding temperature, the necessary pressure on the contact surfaces can be achieved. Considering that AISI 321–Ti-6Al-4V joints will operate under cryogenic temperatures and internal pressure, compressive stresses will arise in the structure, increasing the joint strength. Thus, the second option for producing a diffusion bond is more rational. The copper interlayer has a small thickness of 0.1 mm and, under pressure, deforms to fill the irregularities on the contact surfaces, with minimal impact on the contact pressure.

This diffusion bonding scheme for Ti-6Al-4V titanium alloy and AISI 321 stainless steel components was considered in [20]. The authors developed a physical and mathematical model of diffusion bonding of dissimilar materials along contact surfaces. The calculations were made without considering the copper foil interlayer. Numerical experiments defined the main geometric parameters and material properties to ensure the required bonding conditions. Based on the results of the work, one can select optimal dimensions and physical, mechanical, and thermophysical properties of materials for a bimetallic structure and perform calculations for production of bimetallic adapters used in aerospace components.

**Figure 1:**
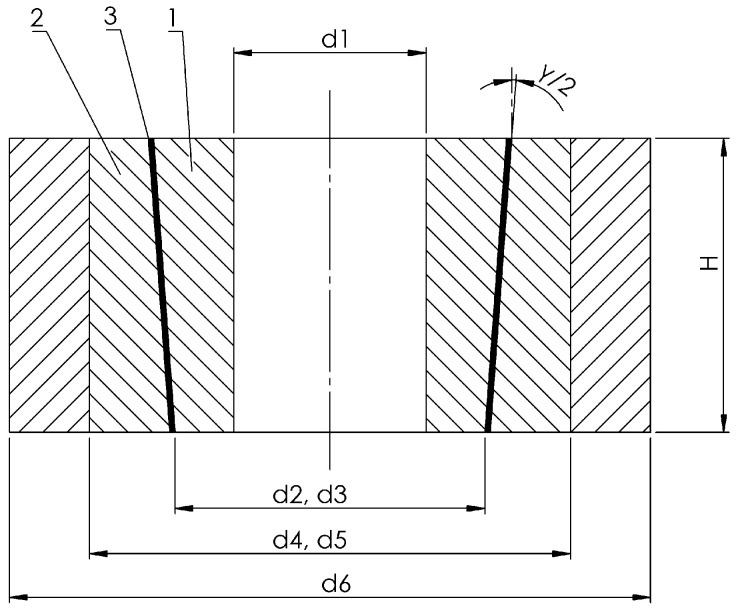
Diagram of the welded joint with the external sleeve: 1—Ti-6Al-4V alloy part; 2—AISI 321 stainless steel part; 3—Cu layer.

Experimental samples were fabricated with the following geometric characteristics (Figure 1): *d*_1_ = 6 mm; *d*_2_ *= d*_3_ = 11 mm; *d*_4_ = *d*_5_ = 18 mm; *H* = 20 mm; and γ = 1.438°. This enabled the following diffusion bonding parameters: *p* = 5 × 10^6^ Pa, and *T* = 1123 K (850 °C).

The bonded materials have different physico-mechanical and thermophysical properties. To achieve the necessary contact pressure at the bonding temperature, the inner part was made of Ti-6Al-4V alloy, and the outer part of AISI 321 stainless steel. A 0.1 mm thick copper foil was placed between them. The assembly was enclosed in an external sleeve made of a refractory metal with specific physico-mechanical properties. The joint was then sealed with a special coating, heated, and held in a muffle furnace in an air atmosphere, which does not require gas atmosphere control and is more cost-effective than furnaces with special heating environments [13].

The samples were bonded at a temperature of *T* = 1123 K (850 °C) with holding times of *t* = 30 min and *t* = 15 min. After welding, the samples were cut longitudinally and subjected to microstructural and micro-X-ray spectral analysis.

The samples were cut along the axis into segments. Cross-sectional metallographic specimens were prepared. The surfaces intended for microstructural examination were treated using abrasive paper, a paper wheel with diamond pastes, and cloth moistened with a chromium oxide suspension. Final polishing was carried out on lumber cloth. The rotation speed of the polishing disk did not exceed 800–1200 rpm. Then the samples were electrolytically etched in a 5% oxalic acid solution to reveal the structure of the transition zone from the stainless steel side. A second etchant consisting of equal parts nitric acid, hydrofluoric acid, and glycerine was used to reveal the titanium alloy structure and weld zone. The microstructure of the transition zone was studied on etched and unetched samples using an MIM-8M (made in the USSR; Asma-pribor, LLC, Moscow, Russia) metallographic microscope at magnifications from ×100 to ×1500.

The micro-X-ray spectral analysis method is based on focusing a high-energy electron beam into a micron-sized probe using an electromagnetic lens system. The beam is directed at a selected area of the sample, observed under an optical microscope at 400–600× magnification. When the electrons strike the sample, they excite X-ray radiation in the analyzed region. This radiation is dispersed into a spectrum using crystal analyzers and recorded by detectors. Micro-X-ray spectral analysis was performed using a Cameca MS-46 (made in France; AMETEK, Inc., Toulouse, France) microanalyzer.

**Figure 2:**
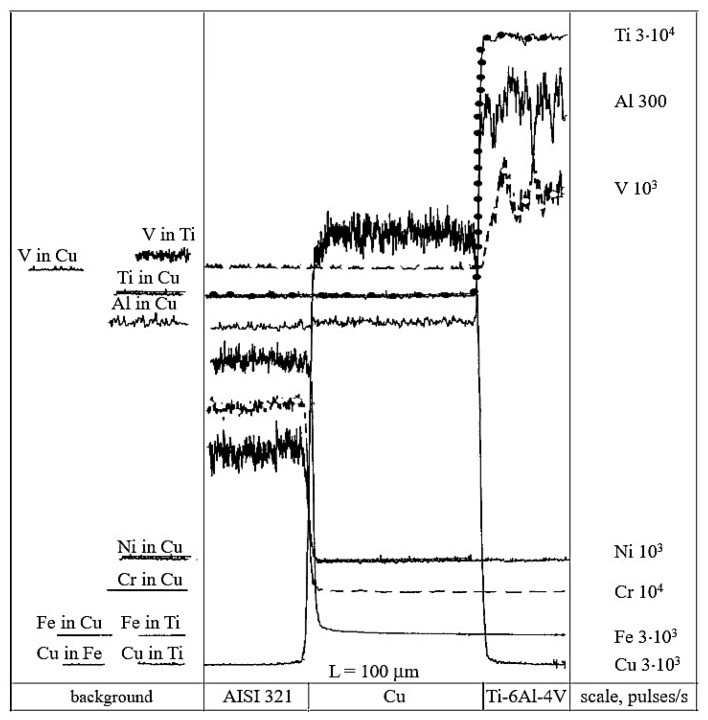
X-ray spectral analysis of cold contact of AISI 321–Cu–Ti-6Al-4V.

To clearly identify the diffusion components in the joints, a methodological approach was employed—linear scanning of a non-diffusion cold contact between AISI 321–Cu–Ti-6Al-4V samples (Figure 2). The starting point for determining mutual diffusion was identified based on the changes in the intensity of element radiation. This method accounts for both instrumental and methodological errors, which, for the studied joints, range from 2 to 6 µm.

Based on the data obtained from micro-X-ray analysis, concentration profiles of the main and alloying elements in the joined materials will be presented in the relevant graphs. The concentrations were determined as a first approximation, without corrections for atomic number and fluorescence.

The diffusion coefficients of the elements were determined according to the method described in [21], ignoring the concentration dependence of the diffusion coefficient (Equation (1)):

(1) $$\frac{C\left(x,t\right)}{C_{0}}=1-\Phi (\omega )$$

where

*x* is the diffusion depth (m);

*t* is the annealing time (s);

*C*(*x*,*t*) is the concentration of direction *x* (%);

*C*_0_ is the concentration value at the initial time point (%);

*D* is the diffusion coefficient (m^2^/s);

Φ(ω) is Crump’s transcendental function, where *ω* is the argument defined by the Equation (2):

(2) $$\omega =\frac{x}{2\sqrt{D\times t}}$$

The AISI 321–Cu–Ti-6Al-4V welded joints have a cylindrical structure, which prevents the use of standard strength testing methods. Therefore, the joints were tested in shear. The samples were cut into rings perpendicular to the axis. Then, the inner ring was pressed out using a hydraulic press with numerical indicated force (TMAX-DYP-60TFS, Xiamen Tmax Battery Equipments Limited, Xiamen, China), and the force required was recorded. Through the use of this force and the area of the contact surfaces, the shear strength of the joint was calculated and compared with reference shear strength values of the base materials and the copper interlayer. Allowable shear strength values for the Ti-6Al-4V alloy, AISI 321 stainless steel, and copper are given in Table 2, Table 5 and Table 8.

The following methods were used in the study of AISI 321–Cu–Ti-6Al-4V welded joints obtained by diffusion bonding along developed conical surfaces:

- Calculation methods—to determine the diffusion coefficients of the elements.
- Metallographic analysis—to study the dimensions and composition of the transition zone in the AISI 321–Cu–Ti-6Al-4V joint.
- Micro-X-ray spectral analysis—to determine the diffusion depth of base and alloying elements of the alloys.
- Mechanical shear testing of the joints.

## 3. Results and Discussion

### 3.1. Determination of the Diffusion Depth of Base and Alloying Elements

**Figure 3:**
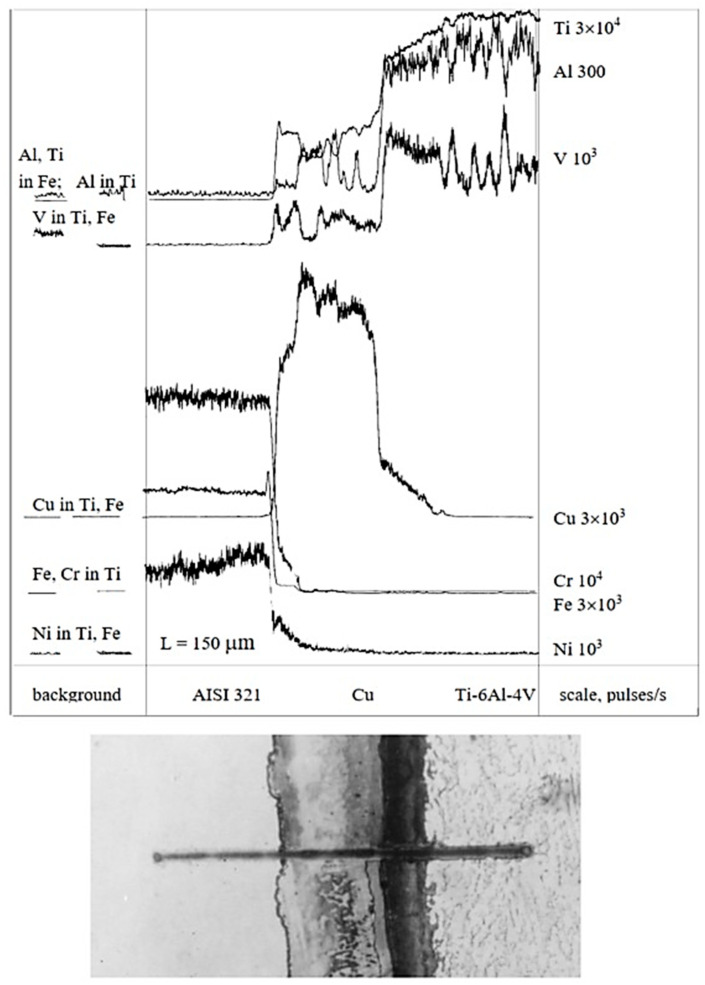
X-ray spectral analysis in the joint AISI 321–Cu–Ti-6Al-4V, *t* = 15 min (×450, ×1.5).

**Figure 4:**
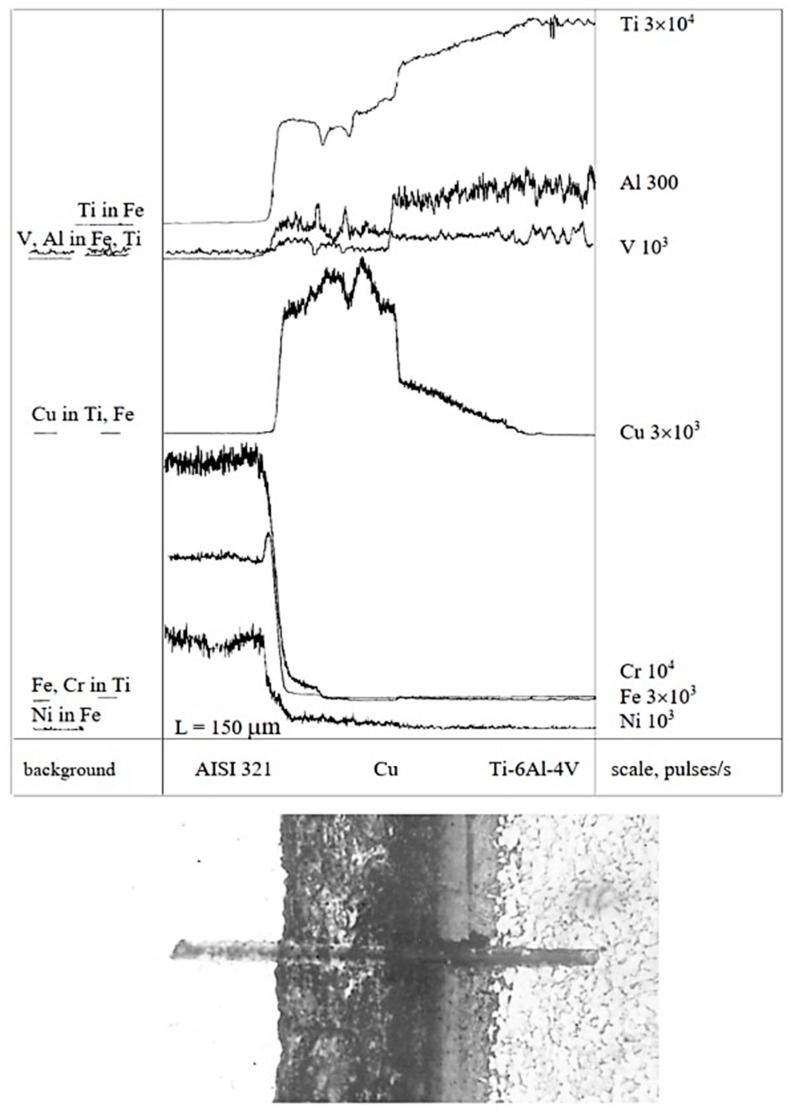
X-ray spectral analysis in the joint AISI 321–Cu–Ti-6Al-4V, *t* = 30 min (×450, ×1.5).

**Figure 5:**
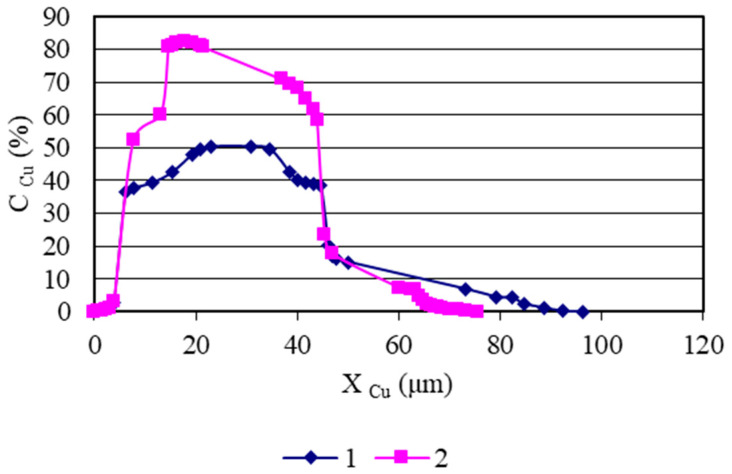
Concentration curves of copper in the diffusion zone of AISI 321–Cu–Ti-6Al-4V joints: 1—sample group welded at *t* = 30 min; 2—sample group welded at *t* = 15 min.

**Figure 6:**
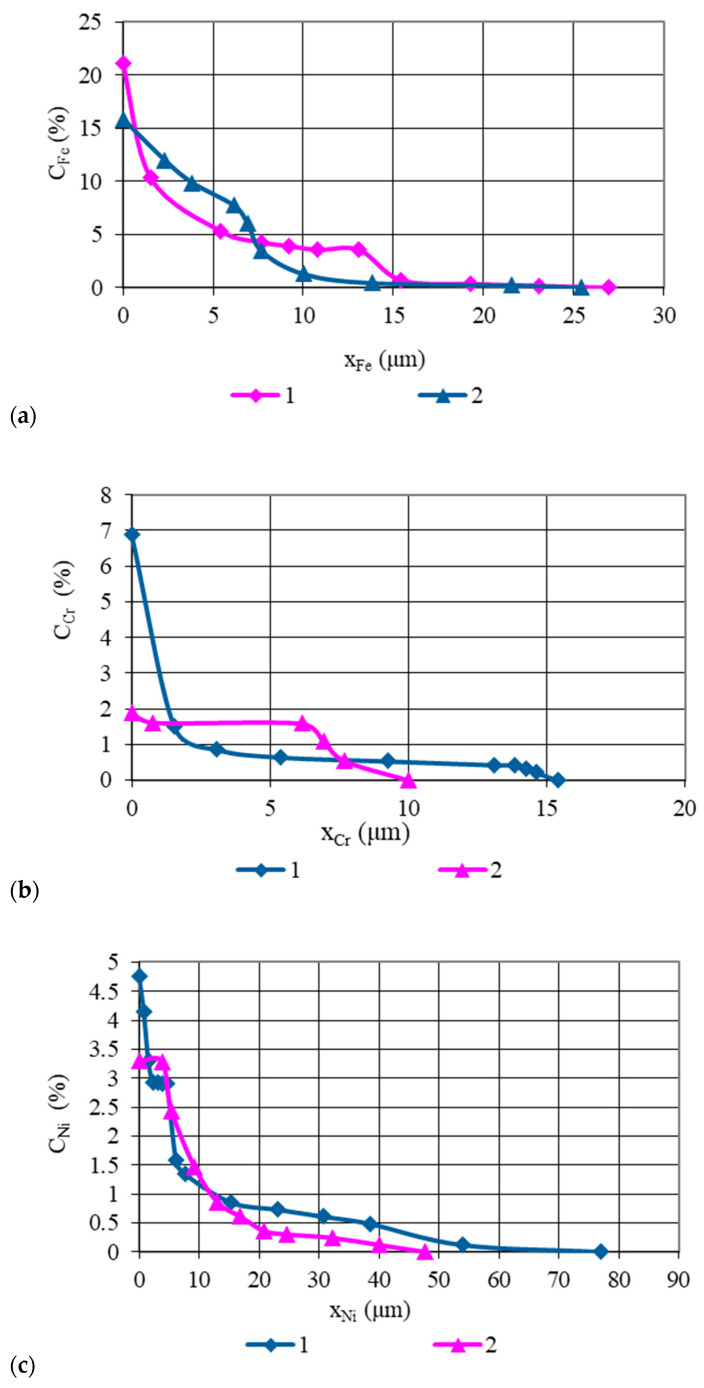
Concentration curves of the base and alloying elements of stainless steel AISI 321 in the diffusion zone of AISI 321–Cu–Ti-6Al-4V joints, welded at *T* = 1123 K (850 °C): 1—sample group 1 welded at *t* = 30 min; 2—sample group 2 welded at *t* = 15 min; (**a**)—Fe distribution; (**b**)—Cr distribution; (**c**)—Ni distribution.

**Figure 7:**
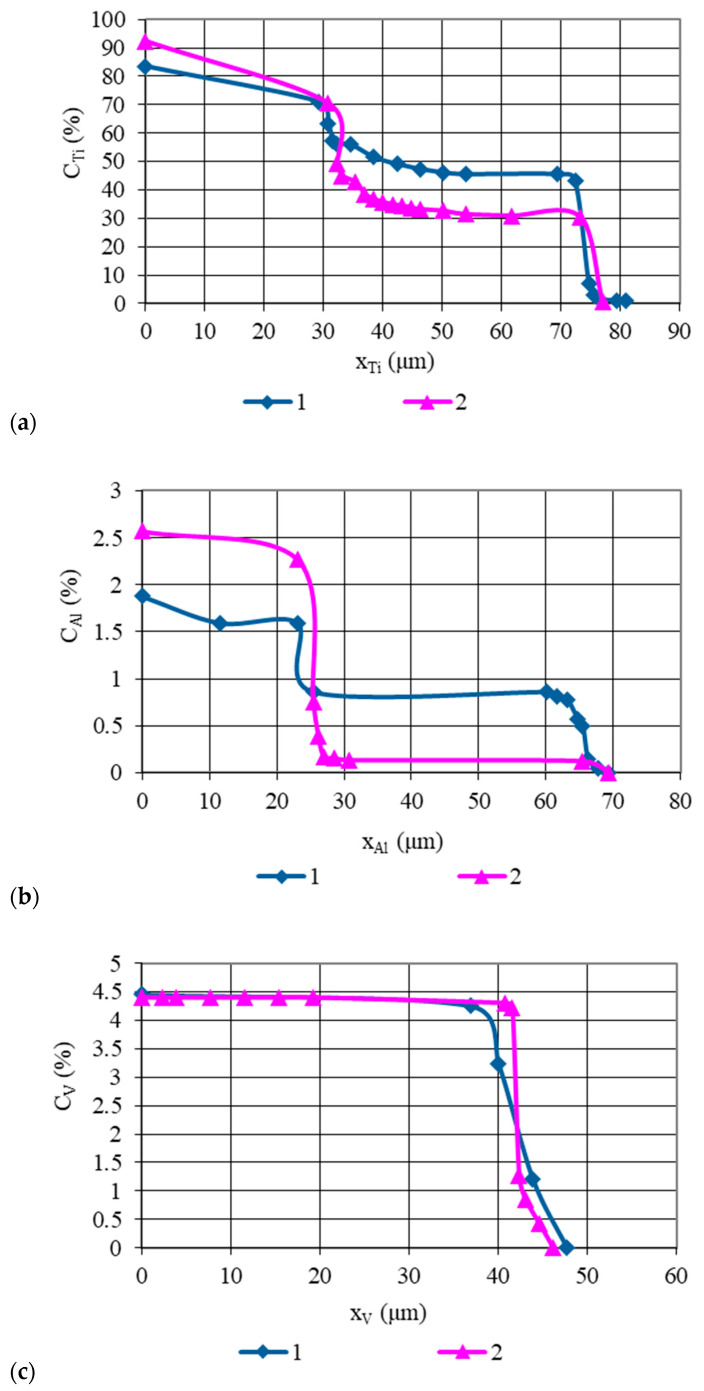
Concentration curves of the base and alloying elements of titanium alloy Ti-6Al-4V in the diffusion zone of AISI 321–Cu–Ti-6Al-4V joints, welded at *T* = 1123 K (850 °C): 1—sample group 1 welded at *t* = 30 min; 2—sample group 2 welded at *t* = 15 min; (**a**)—Ti distribution; (**b**)—Al distribution; (**c**)—V distribution.

Concentration curves of copper, as well as those of the base and alloying elements of AISI 321 stainless steel and the Ti-6Al-4V titanium alloy, obtained from micro-X-ray analysis and illustrated in Figure 3, Figure 4, Figure 5, Figure 6 and Figure 7, indicate that the maximum mutual diffusion occurs at the titanium alloy interface. The diffusion characteristics are quantitatively presented in Table 10, which provides the diffusion depths of the base and alloying elements within the AISI 321–Cu–Ti-6Al-4V joint.

Titanium, aluminum, and vanadium diffuse through the entire copper interlayer with average concentrations of C_Ti_ = 46%, C_Al_ = 0.85%, and *C_V_* = 4.4% at *t* = 30 min and C_Ti_ = 32%, C_Al_ = 0.12%, and *C_V_* = 4.4% at *t* = 15 min. On the stainless steel side, the diffusion depth of iron and chromium into copper is limited and amounts to 19.25 µm and 15.4 µm at *t* = 30 min, and 13.86 µm and 10 µm at *t* = 15 min (Table 7). Nickel, however, diffuses through the entire copper interlayer, with its concentration decreasing to zero at a depth of 77 µm at *t* = 30 min and 47.74 µm at *t* = 15 min.

### 3.2. Determination of Diffusion Coefficients of Elements and the Diffusion Mechanism

Based on the concentration curves obtained from micro-X-ray analysis, the data were processed using Equation (1), and the diffusion coefficients *D_p_* were subsequently calculated using Equation (2). These calculated diffusion coefficients, together with reference bulk diffusion coefficients *D_t_*, are presented in Table 11, which provides a comparative overview of the diffusion behavior of elements in the AISI 321–Cu–Ti-6Al-4V joint.

**Figure 8:**
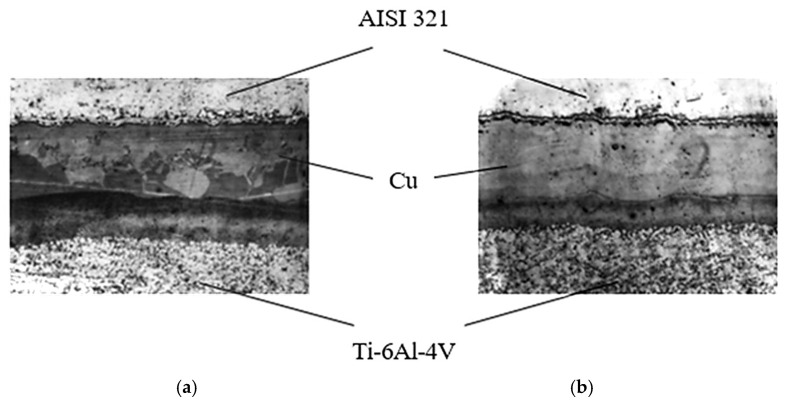
Structure of AISI 321–Cu–Ti-6Al-4V welded joints: (**a**)—holding time *t* = 30 min; (**b**)—holding time *t* = 15 min (×200, ×1.5).

The structure of AISI 321–Cu–Ti-6Al-4V welded joints is presented in Figure 8, illustrating the effect of different holding times: (a) 30 min and (b) 15 min (magnification ×200, scale ×1.5).

Thermodynamic analysis of the binary Cu–Ti phase diagram and the ternary Cu–Fe–Ti system, and microstructural characterization of AISI 321–Cu–Ti-6Al-4V welded joints allow us to assume formation of a liquid phase at the welding temperature [22]. This liquid phase, observed predominantly on the stainless steel side at approximately one-third of the transition zone (see Figure 8), manifests as regions of re-solidified copper-based alloy.

### 3.3. Determination of Mechanical Properties

Based on the evaluation of shear tests conducted on AISI 321–Cu–Ti-6Al-4V welded joints, it was determined that halving the holding time resulted in a 6% reduction in joint strength—specifically, to 250 MPa for *t* = 30 min and 235 MPa for *t* = 15 min. Bending tests performed at a 90° angle did not cause delamination or material failure. The joints were also subjected to internal pressure of 28 MPa across a temperature range of 223 to 273 K. The results of the pressure tests confirmed that the joints successfully withstood the specified loads.

## 4. Discussion of the Results of the Study of AISI 321–Cu–Ti-6Al-4V Diffusion-Welded Joints with Developed Conical Contact Surfaces

From the concentration curves shown in Figure 2, Figure 3 and Figure 4, the diffusion depth of the base and alloying elements in the AISI 321–Cu–Ti-6Al-4V joint was determined. The distribution of nickel and aluminum in copper is explained by the equilibrium phase diagrams of the Cu–Ni and Al–Cu systems [22], which show solid solution series based on different compounds. In Cr–Cu and Cu–V systems, no intermetallic or intermediate phases are formed. The solubility of chromium and vanadium in copper is very limited (*C_V_* = 0.1 at.%, *C_Cr_* = 0.024 at.% at 673 K (400 °C)) and significantly lower than the concentrations observed in the transition zone of the joint. This would typically lead to immiscibility, segregation, and limited diffusion in the joint. However, the significant diffusion depth of Cr and V and their high concentrations indicate a discrepancy with the equilibrium binary diagrams of Cr–Cu and Cu–V. This deviation is explained by the dislocation diffusion mechanism, which enhances material penetration under the deformation and thermal conditions of diffusion welding on enveloping surfaces.

High mutual diffusion of copper and titanium also occurs due to dislocation motion both within and between grains, resulting from assembly and thermal strains during welding. Grain boundary diffusion is clearly visible on the titanium side and likely precedes bulk diffusion (Figure 8). The calculated diffusion coefficients *D_p_* using Equation (1) and Equation (2) exceed the tabulated bulk diffusion coefficients *D_t_* by factors of 10^1^ to 10^7^ (Table 8).

The appearance of a liquid phase (L) in the Cu–Ti system is attributed to two invariant reactions—L = Cu_4_Ti + Cu_2_Ti and L + Cu_3_Ti_2_ = Cu_2_Ti—which occur at 1148 K (875 °C) and 1153 K (880 °C), respectively [14]. The welding temperature was deliberately chosen to be below these temperatures at *T* = 1123 K (850 °C). Nevertheless, a liquid phase forms due to the combined effect of diffusing elements in the ternary Cu–Fe–Ti system and the lowering of the solidus temperature with increasing Fe and Ti concentrations [16].

Shear tests of AISI 321–Cu–Ti-6Al-4V joints showed that reducing the holding time slightly decreases joint strength. The strength of the obtained joints is higher than that of the copper interlayer (*P* = 180 MPa) but lower than the strength of AISI 321 stainless steel (*P* = 330 MPa). Thus, the joint strength is limited by the properties of the transition zone formed during welding. Additional tests under bending and internal pressure of 28 MPa at temperatures from 223 to 273 K demonstrated the joints’ reliability under service loads. This is explained by the strength provided by the entire two-layer structure due to the difference in the coefficients of thermal expansion of the titanium alloy and stainless steel, and the sealing ensured by the vacuum-tight diffusion bond. Therefore, the achieved joint strength of *P* = 250 MPa is sufficient under the specified operating conditions.

The obtained results on the diffusion depth of elements, calculated diffusion coefficients, and mechanical tests allow for the following recommendations for diffusion welding to be made:

- Welding scheme: developed conical contact surfaces.
- Welding of AISI 321 stainless steel and Ti-6Al-4V titanium alloy should be performed using an intermediate copper layer.
- Welding should be performed in a die assembly with full sealing in furnaces with an air atmosphere.
- Recommended welding parameters: *T* = 1123 K (850 °C) and *t* = 30 min.

The developed technology is proposed for manufacturing bimetallic adapters from Ti-6Al-4V and AISI 321 stainless steel used to connect high-pressure titanium tanks to stainless steel pipelines.

The results can be used to manufacture Ti-6Al-4V and AISI 321 adapters operating under cryogenic temperatures. Higher temperatures may result in radial tensile stresses leading to joint failure. To obtain joints operating in a different temperature range, it is necessary to consider the influence of thermophysical and physico-mechanical properties on joint performance.

A limitation of this study is that joints with only one set of geometric parameters and materials were investigated. Future research is planned to expand the range of materials and applications of dissimilar metal joints with developed conical contact surfaces.

## 5. Conclusions

1. Microstructural and micro-X-ray spectral analyses allowed the determination of the diffusion depth of base and alloying elements in the AISI 321–Cu–Ti-6Al-4V joint. High mutual diffusion of copper and titanium and elevated concentrations of Cr and V in copper were observed. Such behavior is not characteristic of joints obtained by previously described technologies.
2. Based on diffusion depth, the diffusion coefficients of elements in the AISI 321–Cu–Ti-6Al-4V joint were determined. It was found that the calculated diffusion coefficients exceed tabulated bulk values by factors of 10^1^ to 10^7^. This is attributed to the dislocation diffusion mechanism inside and outside grain boundaries due to assembly and thermal strains during welding. A liquid copper phase was also found to form in the transition zone during welding, promoting active diffusion and strong joint formation.
3. Shear tests of AISI 321–Cu–Ti-6Al-4V joints revealed a strength decrease of 6% when the holding time was halved (250 MPa and 235 MPa at *t* = 30 and 15 min, respectively). The strength trend is consistent with other studies. However, shear strength can only be compared to reference values, as most studies report tensile strength. Other tests, such as bending and service loading, confirmed that the bimetallic joints of AISI 321–Cu–Ti-6Al-4V have sufficient strength and sealing capability.

Recommendations for diffusion welding technology of AISI 321–Cu–Ti-6Al-4V joints with developed conical contact surfaces were developed, with the following recommended parameters: *T* = 1123 K (850 °C) and *t* = 30 min.

The AISI 321–Cu-ETP–Ti-6Al-4V joints, obtained by diffusion bonding, are recommended for manufacturing bimetallic adapters. These adapters, with suitable mechanical and thermal properties, are designed to connect high-pressure titanium vessels to stainless steel pipelines in cryogenic conditions.

## Figures and Tables

**Table 1:** Chemical composition of titanium alloy Ti-6Al-4V [14].

Alloy Components	Mass Fraction of the Element (%)
Al	6.0
Fe	Max. 0.25
O	Max. 0.2
Ti	90.0
V	4.0

**Table 2:** Physico-mechanical properties of titanium alloy Ti-6Al-4V at room temperature [14].

Properties	Value
Density	4430 kg/m^3^
Tensile Strength, Ultimate	950 MPa
Tensile Strength, Yield	880 MPa
Shear Strength	550 MPa
Elongation at Break	14%
Elastic Modulus	113.8 GPa
Poisson’s Ratio	0.342

**Table 3:** Thermal and physical properties of titanium alloy Ti-6Al-4V [14].

Properties	Value
CTE, linear 293 K (20 °C)	8.6 × 10^−6^ m/m K
CTE, linear 523 K (250 °C)	9.2 × 10^−6^ m/m K
CTE, linear 773 K (500 °C)	9.7 × 10^−6^ m/m K
Melting point	1877–1933 K (1604–1660 °C)
Solidus	1877 K (1604 °C)
Liquidus	1933 K (1660 °C)

**Table 4:** Chemical composition of stainless steel AISI 321 [16].

Alloy Components	Mass Fraction of the Element (%)
C	0.08
Mn	2.0
Si	1.0
Cr	18.0
Ni	11.0
P	0.045
S	0.03
N	0.1
Fe	68.0
Ti	0.15

**Table 5:** Physico-mechanical properties of stainless steel AISI 321 at room temperature [16].

Properties	Value
Density	8000 kg/m^3^
Tensile Strength, Ultimate	620 MPa
Tensile Strength, Yield	240 MPa
Shear Strength	330 MPa
Elongation at Break	45%
Elastic Modulus	193 GPa
Poisson’s Ratio	0.3

**Table 6:** Thermal and physical properties of AISI 321 [16].

Properties	Value
CTE, linear 293 K (20 °C)	16.7 × 10^−6^ m/m K
CTE, linear 523 K (250 °C)	17.1 × 10^−6^ m/m K
CTE, linear 773 K (500 °C)	18.5 × 10^−6^ m/m K
Melting point	20.5 × 10^−6^ m/m K
Solidus	1673–1698 K (1400–1425 °C)
Liquidus	1673 K (1400 °C)
Properties	1698 K (1425 °C)

**Table 7:** Chemical composition of copper Cu-ETP [19].

Alloy Components	Mass Fraction of the Element (%)
Cu	Min. 99.9
O	Max. 0.04
Bi	Max. 0.0005
Pb	Max. 0.005

**Table 8:** Physico-mechanical properties of copper Cu-ETP at room temperature [19].

Properties	Value
Density	8900 kg/m^3^
Tensile Strength, Ultimate	220–260 MPa
Tensile Strength, Yield	140 MPa
Shear Strength	180 MPa
Elongation at Break	33%
Elastic Modulus	127 GPa

**Table 9:** Thermophysical properties of copper Cu-ETP [19].

Properties	Value
CTE, linear 293–573 K (20–300 °C)	17.7 × 10^−6^ m/m K
Melting point	1356 K (1083 °C)

**Table 10:** Diffusion depth of base and alloying elements in the joint AISI 321–Cu–Ti-6Al-4V.

Part Material, Main and Alloying Elements	Diffusion Depth During Welding (10^−6^ m)	
	*t* = 15 min	*t* = 30 min
Stainless steel AISI 321		
Fe	13.86	19.25
Cr	10.0	15.4
Ni	47.74	77.0
Cu		
Cu in Fe	3.85	3.85
Cu in Ti	28.5	45.43
Titanium alloy Ti-6AL-4V		
Ti	46.2	50.0
Al	42.35	43.89
V	46.5	48.0

**Table 11:** Diffusion coefficients of elements in the AISI 321–Cu–Ti-6Al-4V joint [21].

Part Material, Main and Alloying Elements	Diffusion Depth During Welding (10^−4^ m^2^/s)		
	Sample Group 1 *t* = 30 min	Sample Group 2 *t* = 15 min	*D_t_*
	*D_p_*	*D_p_*	
Stainless steel AISI 321			
Fe	3 × 10^−10^	1.4 × 10^−10^	1 × 10^−11^
Cr	4.3 × 10^−10^	6.2 × 10^−8^	1 × 10^−13^
Ni	1.9 × 10^−8^	1.3 × 10^−7^	7.6 × 10^−14^
Cu			
Cu in Fe	6.3 × 10^−12^	4.6 × 10^−12^	4.1 × 10^−12^
Cu in Ti	2.9 × 10^−9^	2 × 10^−9^	1.5 × 10^−11^
Titanium alloy Ti-6AL-4V			
Ti	4.2 × 10^−7^	7.9 × 10^−8^	1 × 10^−13^
Al	1.2 × 10^−7^	1.7 × 10^−7^	1.7 × 10^−9^
V	4.7 × 10^−7^	2.1 × 10^−6^	1 × 10^−13^

## Data Availability

The original contributions presented in this study are included in the article. Further inquiries can be directed to the corresponding author.
